# Real-world Data to Generate Evidence About Healthcare Interventions: The Application of an Ethics Framework for Big Data in Health and Research

**DOI:** 10.1007/s41649-019-00095-1

**Published:** 2019-09-04

**Authors:** Wendy Lipworth

**Affiliations:** Sydney Health Ethics, Faculty of Medicine and Health, The University of Sydney, Sydney, Australia

**Keywords:** Big Data, Real-world evidence, Registries, Conflict of interest, Integrity

## Abstract

It is increasingly recognised that evidence generated using “real-world data” (RWD) is crucial for assessing the safety and effectiveness of health-related interventions. This, however, raises a number of issues, including those related to (1) the quality of RWD, and of the scientific methods used to generate evidence from it, and (2) the potential for those gathering and using RWD be driven by commercial, political, professional or personal self-interest. This article is an application of the framework presented in this issue of ABR (Xafis et al. 2019). Please refer to that article for more information on how this framework is to be used, including a full explanation of the key values involved and the balancing approach used in the case study at the end to demonstrate how those generating or using RWD can make use of values when deliberating about ethical issues.

## Background

This domain will illustrate the ethical and governance challenges that arise in the use of “real-world data”. The phrase “real-world data” refers here to data that is collected outside the laboratory or conventional randomised controlled trials (RCTs). Real-world data (RWD) is used in a number of fields including:

- Basic science, where RWD can be used to inform the understanding of disease and the generation of disease taxonomies, assist with the identification of therapeutic targets and biomarkers and guide the targeting of therapies to those who are most likely to respond;
- Public health, where RWD has long been used for disease surveillance and population health (epidemiological) research; and
- Clinical research, where RWD may be used not only to facilitate RCTs (e.g. by aiding recruitment) but also:
  - as data sources for trials that aim to capture the effects of interventions in heterogeneous populations receiving routine healthcare, and that integrate the use of RWD (e.g. large simple trials, pragmatic trials); or
  - to generate observational data about the safety and effectiveness of healthcare interventions (e.g. case series, case-control and cohort studies).

In this domain, the focus will be on the use of RWD to generate real-world evidence (henceforth RWE) about healthcare interventions, which is then used to guide their regulation, financing or clinical use, as well as broader health service design.

RWD can be collected from a number of different sources. It may be generated specifically for research, surveillance or monitoring purposes. Alternatively, it might be derived from other “routinely collected” real-world datasets including: clinical, public health and health administrative datasets such as electronic health records, pharmacy dispensing data and insurance claims data; patient-generated datasets, such as personal health and activity monitoring tools (mobile health), health-related websites and social media platforms; and non-health datasets such as credit card or internet activity data, census data, employment data and general social media.

Collections of RWD are often “big”, either in their own right or because they are linked together to provide a richer picture of the safety, effectiveness or cost-effectiveness of interventions. For example, it might be necessary to link together information about clinical outcomes (from electronic health records), dispensing data (from pharmacy databases) and insurance claims (from administrative databases) in order to determine whether a particular intervention is effective, whether it is affordable for patients and whether it represents “value for money”.

## Two Issues Raised by the Use of Real-world Data

It is increasingly recognised that RWE is crucial for assessing the safety and effectiveness of interventions that are not well suited to being tested in RCTs (for example surgical procedures and health service level interventions) and for supplementing the—always limited—information that can be generated from RCTs. There are, however, several important issues that need to be considered when using RWD to generate RWE. In this domain, we will explore two key issues:

- The quality of RWD, and of the scientific methods used to generate RWE from it, and
- The potential for those gathering and using RWD and RWE to be driven by commercial, political, professional or personal self-interest and, closely related to this, the potential limitations of self-governance.

Both of these issues arise in large part because RWD is often produced in one space (e.g. clinical practice, health administration, social media) and then used in another, which creates both scientific and governance challenges.

### The Quality of Real-world Data and the Scientific Methods Used to Generate Real-world Evidence

There are numerous technical and methodological challenges associated with using RWD to generate RWE. Broadly speaking, these challenges fit into two categories: those related to data quality and those related to scientific methodology.

With respect to data quality, it is important to bear in mind RWD is often collected for clinical or administrative purposes by people not trained in data standards or scientific methodologies, and then used to generate RWE. It is therefore often incomplete or slanted towards particular patient groups, unstandardised, unstructured and not comparable among patients or across time.

With respect to research methodology, even large observational studies are not fully generalisable (i.e. they do not necessarily address this limitation of RCTs). And unlike RCTs, observational studies do not have mechanisms—such as randomisation and blinding—built into them to deal with bias and confounding. Importantly, simply collecting or linking together larger amounts of RWD does not solve these problems and may, indeed, exacerbate them. This is because the size of Big Data collections makes it possible to address a large range of questions—often without a clear hypothesis—and to adjust for an enormous range of variables. Given these risks, the analysis of RWD requires special vigilance to avoid data users drawing unjustified conclusions (based on spurious correlations) about the causal nature of associations observed.

**Table:** 

*Examples: Pharmacovigilance and safety audits* Medicines regulators are responsible not only for deciding which products should be approved for marketing, but also for detecting and responding to safety concerns once medicines are on the market (pharmacovigilance). To achieve this, regulators are increasingly supplementing information derived from standard RCTs with information from observational studies, clinical registries, audits, electronic medical records, insurance claims data and other—often networked—sources of RWD. New techniques such as machine learning, cognitive computing and natural language processing are also being developed in order maximise the utility of RWD for pharmacovigilance. RWD adds considerable value to pharmacovigilance efforts—for example, it seems likely that had such systems been available in the past, harmful drugs such as Vioxx™ (rofecoxib), would have been withdrawn from the market more quickly than they were (Coloma et al. 2013). However, such systems are subject to all the data quality and methodological limitations described above. Simply making the RWD “bigger” does not necessarily solve these problems because conflicting conclusions about safety can be drawn from different datasets (Melloni et al. 2015), and large networked datasets might at times generate enormous numbers of potential safety signals, many of which will subsequently prove to be false. Distinguishing valid from invalid signals can be time consuming, and false causal inferences can lead to the loss of effective interventions. The difficulty of making sense of “real-world” safety signals is evident in the story of rosiglitazone—a drug used to treat type 2 diabetes. After rosiglitazone was approved for marketing, evidence emerged from meta-analyses of RCTs suggesting an increased risk of myocardial ischaemia. Subsequent RWE generated through two independent observational cohort studies failed to reveal an association, which was interpreted at the time as evidence of the limitations of observational studies (Rosen 2007). RWE from health utilisation databases subsequently emerged that suggested that there was in fact a significant risk, and this time the real-world evidence (along with further RCT evidence) was used to justify restrictions on prescribing in the USA and Canada and removal of rosiglitazone from the European market (Rosen 2010). Even now, however, there is controversy not only about the quality of the RCTs and meta-analyses that led to the restriction or removal of rosiglitazone (Stone et al. 2015) but also about the role of RWE—particularly that collected from health utilisation databases—in regulatory decision-making (Rawson 2014). What is noteworthy about these cases is that both RCTs and observational studies were trusted and mistrusted at various times and none of these assessments was obviously wrong—which underscores the complexity of evidence generation when multiple methods can be used to answer similar questions. Another case that demonstrates the challenges of using RWD to assess the safety of healthcare interventions was that of transvaginal mesh (TVM) for pelvic organ prolapse. After safety concerns emerged about the use of TVM for this indication, a series of retrospective reviews were conducted. These reviews were heavily criticised for excluding vital data sources, thus underestimating the number and severity of adverse events. For example, the UK TVM audit was based solely on Hospital Episodes Statistics (HES), and therefore excluded private patients and outpatients (Wise 2018). The reviews were also criticised for failing to provide any context for adverse events. For example, the UK audit stated the number of women (in the public hospital system) who required additional surgery following surgery using transvaginal mesh—but failed to provide any insight as to why additional surgery was required (Royal College of Obstetricians and Gynaecologists 2018). These problems stemmed directly from the reliance upon RWD that had not been collected primarily for research or quality assurance purposes. Interestingly, one of the suggested remedies for this situation was to replace one form of RWE generation (retrospective audits) with another (a prospective registry) (Rimmer 2018).	

### Influence, Conflict of Interest and the Limitations of Self-governance

As is the case with any kind of biomedical research, it is crucial that those gathering and analysing RWD, and making use of evidence generated from this data, are motivated by the desire to enhance the quality, integrity and efficiency of biomedical science and not (solely) by their own interests. Research using RWD is, however, funded, conducted, accessed and translated into policy and practice by people with a wide range of commercial, political, professional and personal interests. Although this is in no way unique to research using RWD (for example, the vast majority of clinical trials are funded by industry and all research is shaped by political, professional and other “agendas”), the potential for commercial, political or other interests to influence this kind of “Big Data” research is greater because standards for data quality are only just beginning to emerge, methods for analysing RWD to generate RWE are still in development, and systems are not fully developed for the registration and reporting of observational research.

This, in turn, underscores the need for appropriate governance of RWD resources and associated research. In this regard, it is noteworthy that although some research processes, such as formal observational research using electronic health records, are subject to external governance by institutional research ethics committees, other uses of RWD are largely self-governed. There are well-known strengths and limitations of self-governance. On the one hand, self-governance draws on ready-made expertise within a field and can be flexible and rapid in its responses to issues as these arise. However, concerns about lack of transparency of such processes, or mechanisms of external accountability, can undermine trust and raise questions about the legitimacy of self-governance. Both strengths and limitations are evident in the processes used to govern RWD resources and the processes used to generate RWE.

**Table:** 

*Example: Stem cell therapy registries* Adult stem cells are used for a variety of clinical indications. The most established of these is bone marrow or stem cell transplantation for haematopeietic (blood-related) malignancies and non-malignant disorders. To improve the transplantation process and increase survival rates, the Medical College of Wisconsin has maintained an International Bone Marrow Transplant Registry for over 40 years. The Centre for International Blood & Marrow Transplant Research (CIBMTR) now curates this database, along with the Autologous Blood and Marrow Transplant Registry, in order to systematically track therapeutic trends and clinical outcomes from over 425,000 patients worldwide (Center for International Blood and Marrow Transplant Research n.d.). At the same time, a number of clinical registries have been established to collect RWE on the outcomes of interventions with adult stem cells that have not been established or accepted as standard clinical care (e.g. to treat various degenerative diseases). Although there is nothing wrong with developing registries to gather data on “unproven” interventions, there are signs that many of these registries—such as that of the International Cellular Medicine Society (ICMS)—have not been designed primarily with the public interest or scientific rigour in mind (Lysaght 2014). For example, these registries are touted as alternatives (rather than supplements) to externally evaluated and regulated research; participating clinicians are free to choose whether or not to submit data to the register; and the data are not freely shared with others. This is in contrast to the CIBMTR which has in place mandates for participating institutions to submit all data in a prospective manner (whether or not they are favourable to their professional, organisational, commercial or political interests), and makes data freely available to researchers worldwide. With respect to governance mechanisms, the CIBMTR shows many signs of deliberate and effective self-governance, including data transparency, regular data audits (in addition to robust validation processes built into data collection systems) and transparency of funding sources. Many adult stem cell registries, in contrast, are non-transparent, with data available only to participating clinicians. They also lack explicit audit mechanisms and funding models are unclear or ad hoc (including asking patients to pay to have their data included in the registries).	

## Some Relevant Values

There are numerous values that can help to guide the conduct and governance of research, surveillance and monitoring using RWD. With respect to the two issues discussed in this domain (scientific quality and competing interests), substantive values of particular relevance are:

- The need to consider the integrity of those involved in the research—that is their ability and willingness to act in accordance with personal and/or accepted scientific and professional values and commitments.
- The need to promote public benefit—in this context as distinct from (only) facilitating commercial, political, professional or organisational imperatives.

And the most relevant procedural values are:

- Reflexivity: those conducting the research need to be alert to their own conflicts of interest and have processes in place for managing them;
- Transparency: the research processes and data therein need to be open to scrutiny and results must be published;
- Accountability: researchers need to be answerable to an ethics committee, a data access committee, a public funder, other researchers and clinicians, patients and the public, and be held responsible for their actions.

## Case Study: Managing Competing and Conflicting Interests in the Generation of Real-world Evidence

In order to illustrate how values might be used to address ethical issues in practice, we offer an outline of a values-based framework for deliberating about a dilemma concerning the use of RWD. This dilemma centres on an issue—industry funding of RWD research—that, although not unique to research using Big Data, plays out in subtly different ways given the methodological and ethical uncertainties associated with Big Data research, and given that patients are not subjected to any physical risks.

A health service that has a well-established electronic medical record system comprising over 500,000 records is approached by a pharmaceutical company that recognises the value of such Big Data in research. The company wants to fund clinician-researchers employed by the health service to examine these records in order to assess a medicine that it sells. The medicine is used to treat hepatitis C and is currently registered and publicly funded for patients with advanced cirrhosis.

The company wants to assess the “real-world” safety and effectiveness of the medicine and compare the outcomes of patients receiving the medicine to those being managed in other ways. They also want to follow the outcomes of untreated patients who have not (yet) developed severe cirrhosis and do not currently qualify for subsidised access to the medicine.

The clinician-researchers will not be paid personally; rather, the money will be paid into academic departmental accounts and used to fund research nurses, conference travel and other organisational expenses. The researchers and the company will both have access to de-identified (coded) patient data but not to identifiable patient records. The clinician-researchers submit an application to the health service’s research ethics committee in order to gain access to the records needed to answer their research questions.

One of the issues that the committee wishes to focus on in detail is how to address the influence of the pharmaceutical company over the research. They undertake the following deliberative process:

1. Stating the problem and distinguishing ethical problems from other (scientific, social, cultural, linguistic and legal) issues

The ethics committee recognises that a key issue here is industry influence over the research—including both direct control by the company and the creation of “conflicts of interest” on the part of the researchers who may feel (consciously or unconsciously) obligated to the company that is funding them, or want to maintain a good relationship with the company in order to receive further funding in the future.

Although the committee is aware that industry-funded research is often of very high quality, they worry that the either company itself or conflicted clinician-researchers might subtly distort the way that the research is designed (e.g. selecting records for inclusion that are more likely to reveal commercially desirable outcomes); conducted (e.g. switching outcome measures without clear justification); interpreted (e.g. emphasising correlations that are commercially favourable, even if these are not clinically significant); and disseminated (e.g. withholding adverse findings from publication).

They also worry that commercial imperatives might impact negatively on the treatment of research participants, including how consent is obtained, confidentiality maintained, and benefits shared with research participants or the community more generally. For example, the company might not use the information it gleans to benefit patients currently receiving the medicine, but rather to subtly encourage “off-label” prescribing or seek an extension of the indications for which the medicine is registered without clear clinical justification. In this case, patients or the health service might need to pay large sums of money for an intervention of questionable clinical necessity.

Although these issues would apply to any industry-funded research project (e.g. an industry-funded randomised trial), the committee notes that the potential for industry influence is greater in this kind of “Big Data” research because data standards and research methods are less developed. The committee worries that this might make it easier for the company to manipulate the conduct, interpretation and dissemination of the research.

Along similar lines, the committee notes that methods and standards for obtaining participants’ consent, protecting their privacy and sharing the benefits of research are less developed in this context than in relation to clinical trials. This increases the risk that participants could be harmed.

Finally, the committee reflects on the fact that its own processes are not as well-established for governing research using RWD as they are for governing clinical trials, and that they might need to develop their own processes and standards, as well as their relationships with other governing entities, such as data custodians.

2. Getting the facts about the problem

The committee makes sure that they understand exactly how, and by whom, the research will be designed and conducted, how it will be governed, and precisely what funding and contractual agreements will be in place between the pharmaceutical company and anyone else who is involved in the research. They recognise that their hospital’s legal department is likely to have less experience with contractual arrangements for research using RWD than for clinical trials, so they take it upon themselves to work collaboratively with the legal department so that everyone is clear about the procedures that will be followed.

3. Considering the relevant values and identifying conflicts among them

The committee determines that substantive value of particular relevance to this issue (i.e. to industry funding of research) are:

- The need to consider the integrity of those involved in the research—in this case with a particular focus on the potential negative impacts of commercial imperatives on their values and commitments; and
- The need to promote public benefit/interest—in this context as distinct from (only) assisting the company to fulfil its commercial goals.

The committee also recognises that there is a potential value-level conflict between their desire to facilitate the generation of knowledge by enabling research that might not otherwise occur, and the need to only promote research that is conducted with a high level of integrity and concern for public benefit.

Recognising that integrity and concern for public benefit cannot be enforced, the committee notes the importance of procedural values such as transparency, accountability and reflexivity (see step 5 for their articulation).

4. Considering as many alternative perspectives as possible

The committee considers the evidence on public and professional views about industry-funded research, and notes that there is generally a high level of support for such research provided that research participants are aware of the funding source and that steps are taken to ensure that research is conducted appropriately, and benefits are shared. They note that that this (qualified) public support for industry-funded research extends to research using electronic medical records and other sources of RWD.

They also recognise that there are no regulatory impediments to allowing industry-funded research—including research using medical records—although their health service does have a policy stating that industry funders must not be directly involved in the analysis or dissemination of research results.

5. Weighing up the relative ethical merit of the different options and making an ethical decision

The committee considers itself to have three main options:

- Allowing the research to proceed as requested
- Not allowing the research to proceed at all if industry is involved
- Allowing the research to proceed, but only if certain conditions are fulfilled

They decide that option 1 is unacceptable because of the risks articulated in step 1 and the risks to both integrity and public benefit. They are also reluctant to follow option 2, as they believe the research (and associated departmental funding) to be of potential benefit to both current and future patients. They therefore decide that option 3—a compromise approach—is most appropriate and approve the study subject to the following requirements in order to promote the values they have identified as being relevant:

- That a scientific review and feasibility assessment will be conducted to ensure that the hospital’s records are “fit for purpose” (i.e. that data will be sufficiently robust to achieve the study’s outcomes);
- That all relevant medical records will be included in the study to avoid selection biases;
- That clinical outcomes will be defined and assessed using widely agreed and objective measures;
- That clinical outcomes will be selected that are likely to benefit patients rather than serve (only) commercial interests;
- That outcomes will only be changed if there is a strong and justification for doing so, the committee will be notified, and these changes will be transparent in subsequent publication;
- That the study will be listed in a public register prior to commencement, along with a detailed protocol;
- That collection, analysis and—importantly—interpretation will be checked by independent researchers who are not funded by industry and who will pay particular attention to the risk of bias in observational research and the difficulties in drawing conclusions from statistically significant associations within “Big Data”;
- That results—whether favourable to the product or not—will be made available in a registry and submitted for publication in order to maximise their public benefit;
- That the researchers will share their de-identified data publicly (or make a strong case for not doing so) so that other researchers can re-analyse it and compare it to the data generated by other studies that might subsequently be conducted;
- That the researchers will disclose their industry funding to patients and in any subsequent presentations and publications;
- That there is an ongoing independent auditing processes in place to ensure that each of the above requirements is met.

6. Communicating the Decision

The committee communicates and documents its decision and puts in place processes to evaluate its outcomes. In particular, it insists that a detailed annual report be provided, which includes clear evidence that each of the above requirements have been met.

Although we have outlined a deliberative process that allowed industry-funded RWD research to proceed, there is no single preferable approach. As with any ethical issue, decisions regarding the funding of RWD research require complex trade-offs among competing values, and there are always multiple ethically justifiable decisions. What matters is how decisions are reached, justified and followed up.

## Conclusion

Real-world evidence is an increasingly important part of the clinical research landscape, and real-world data is needed in order to generate such evidence. Real-world data is, however, often derived from “secondary” sources and is not always fit for purpose when it comes to the generation of real-world evidence. The methods used to analyse rea-world data are also potentially problematic—and the “bigness” of the data can exacerbate these methodological problems. The foregoing technical and scientific issues—combined with a relative lack of experience in governing research using real-world data—renders this research susceptible to commercial, political and professional biases. Those governing real-world data collections and the research used to generate real-world evidence thus need to be alert to both methodological issues and distortions stemming from competing and conflicting interests.
